# Thymic Hyperplasia and Graves Disease: A Nonincidental Association

**DOI:** 10.1210/jcemcr/luad083

**Published:** 2023-09-01

**Authors:** Begoña Pla Peris, Pablo Abellán Galiana, Francisco Javier Maravall Royo, Agustín Ángel Merchante Alfaro

**Affiliations:** Department of Endocrinology and Nutrition, Hospital General Universitario de Castellón, 12004 Castelló de la Plana, Castellón, Spain; Department of Endocrinology and Nutrition, Hospital General Universitario de Castellón, 12004 Castelló de la Plana, Castellón, Spain; Department of Medicine and Surgery, Universidad Cardenal Herrera-CEU, 12006, CEU Universities, Castellón, Spain; Department of Endocrinology and Nutrition, Hospital General Universitario de Castellón, 12004 Castelló de la Plana, Castellón, Spain; Department of Medicine, Jaume I University, 12006, Castelló de la Plana, Castellón, Spain; Department of Endocrinology and Nutrition, Hospital General Universitario de Castellón, 12004 Castelló de la Plana, Castellón, Spain; Department of Medicine, Jaume I University, 12006, Castelló de la Plana, Castellón, Spain

**Keywords:** Graves disease, thymic hyperplasia, autoimmune, mediastinal mass

## Abstract

We present 2 cases referred for evaluation of Graves disease (GD) associated with an incidental mediastinal mass. Chest computed tomography (CT) scans showed a 1.2 × 2.4 × 4.3 cm and a 5.7 × 2.6 × 7 cm thymic enlargement, respectively, consistent with thymic hyperplasia (TH) in the 2 patients. Patient 1 had been assessed by thoracic surgery for the mediastinal mass, and thymectomy had been performed to exclude thymoma, with an anatomopathological diagnosis consistent with thymic hyperplasia. Patient 2 was treated with methimazole. CT scan was repeated after he maintained a euthyroid state, which revealed total regression of the mass. There is a well-documented association between these 2 entities, but it is often underdiagnosed and unrecognized in routine clinal practice. The benign evolution, as evidenced by regression of thymic hyperplasia after resolution of the hyperthyroidism, is characteristic. These cases highlight the importance of recognizing the association of GD and TH and warrant a conservative approach, preventing unnecessary thymic evaluation and surgery.

## Introduction

Graves disease (GD) is an autoimmune disease characterized by the production of thyrotropin receptor (TSHR) antibodies that have a stimulating effect on the thyroid gland. There is a nonincidental and well-documented association between GD and thymic hyperplasia (TH). TH in the setting of GD seems to involve both an immunological pathogenesis and a thyroid hormone-dependent pathogenesis. Nonetheless, this association is often underdiagnosed and unrecognized in routine clinical practice, and the mechanisms behind this association have yet to be thoroughly elucidated. Thymic regression with the resolution of the hyperthyroidism is characteristic. We describe 2 cases of TH in patients with GD. We analyze the clinical course, the causative mechanisms underlying this association, and the required treatment approach, which we believe is crucial to understand in these patients to prevent unnecessary thymic evaluation and surgery.

## Case Presentation

### Case 1

**Figure 1.**
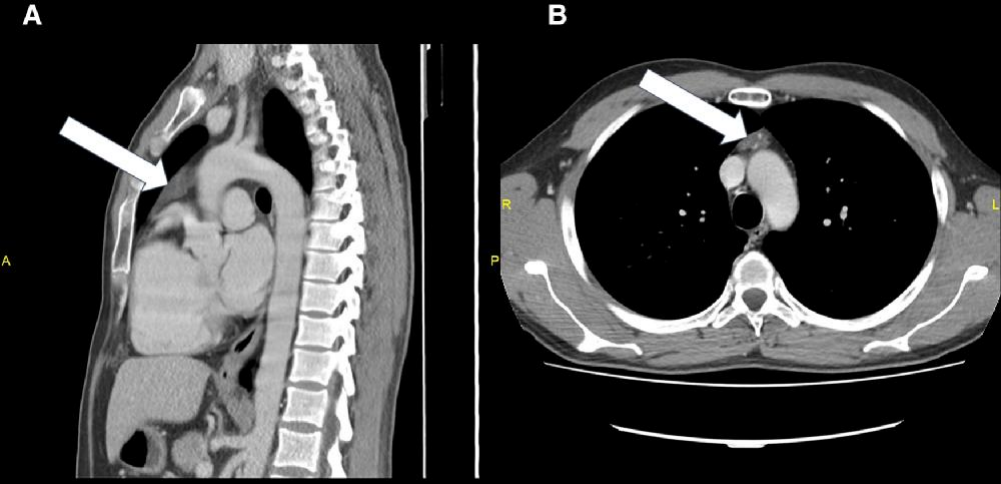
A and B, Computed tomography image at the time of diagnosis showing the enlarged thymus. A, Lateral and B, axial plane views.

A previously healthy 55-year-old man was referred to the endocrinological team for a GD follow-up. Since he had moved residences, a change in hospital assignment was needed. As reflected in reports provided by the hospital of origin, he had been assessed by thoracic surgery for a mediastinal mass. A contrast-enhanced chest computed tomography (CT) scan was performed to evaluate chest pain and palpitations in the setting of GD. This revealed a 1.2 × 2.4 × 4.3 cm solid homogeneous anterosuperior mediastinal mass (Fig. 1A and 1B) without calcification or invasion. The patient had been evaluated by thoracic surgery, and thymectomy had been performed to exclude thymoma, yielding an anatomopathological diagnosis consistent with TH. Given the patient's history, with GD and TH being coincident in time, the diagnosis of GD-associated TH was made.

### Case 2

**Figure 2.**
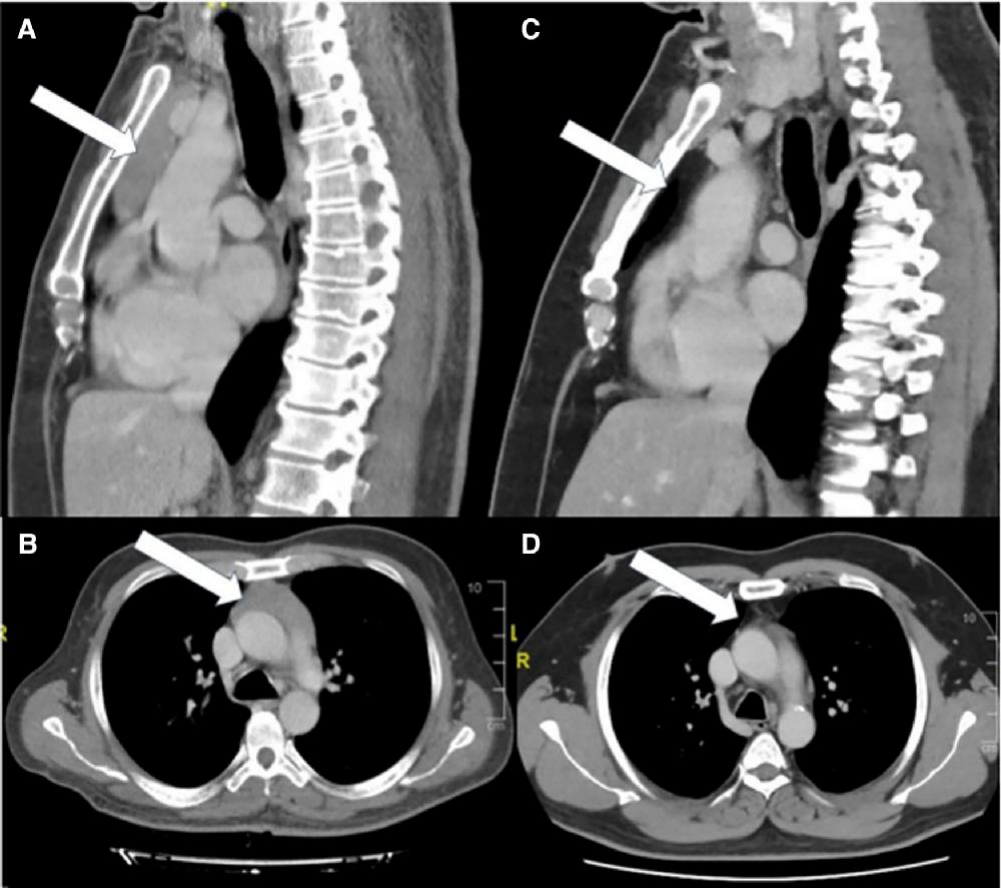
A and B, Initial computed tomography (CT) image at the time of diagnosis showing the enlarged thymus. A, Lateral and B, axial plane views. C and D, CT image after the patient achieved euthyroidism. C, Lateral and D, axial plane views.

We present the case of a 40-year-old man with no personal or family history of interest who was referred for primary hyperthyroidism. On examination, he presented with diffuse goiter, hand tremor, and increased heart rate. Laboratory findings showed TSH less than 0.01 μIU/mL (0.380-5.330 μIU/mL) (0.01 mIU/L [0.380-5.330 mIU/L]) with FT4 4.2 ng/dL (0.54-1.24 ng/dL) (54.05 pmol/L [6.95-15.95 pmol/L]) and positive anti-TSH receptor antibodies (TRAbs) 40 IU/L (reference range < 1.75 IU/L). Autoantibodies against acetylcholine receptor were negative. Thyroid ultrasound showed an enlarged thyroid with increased vascularity. A contrast-enhanced thoracoabdominal CT scan had been performed in the primary care setting because of constitutional symptoms consistent with weight loss, anorexia, and myalgia. It revealed a 5.7 × 2.6 × 7 cm solid anterosuperior mediastinal mass consistent with TH (Fig. 2A and 2B), without calcification or invasion of adjacent structures. No signs or symptoms of myasthenia gravis were noted. A positron emission tomography CT (PET-CT) scan was performed to exclude thymoma, revealing no metabolic activity increase. Based on these findings, the patient was diagnosed with GD and coexisting TH.

## Treatment

### Case 1

At his evaluation in our center, physical examination revealed a homogeneously enlarged thyroid gland without palpable nodules. No signs or symptoms of myasthenia gravis were noted. Medical management included laboratory testing to assess thyroid function, with both normal TSH and free thyroxine (FT4) in the reference range (TSH 1.56 μIU/mL [0.380-5.330 μIU/mL] [1.56 mIU/L; 0.380-5.330 mIU/L]; FT4 0.9 ng/dL [0.54-1.24 ng/dL] [11.58 pmol/L; 6.95-15.95 pmol/L]). Thyroid-stimulating immunoglobulin was 2.4 IU/L (reference range <1.75 IU/L).

### Case 2

Since TH is an uncommon but known manifestation of GD, no further investigations of the thymic mass were conducted, and medical treatment of GD was started with a titration regimen of methimazole and propranolol. A conservative and observational approach was taken to monitor TH.

## Outcome and Follow-up

### Case 1

Treatment with methimazole had been started 6 months earlier, and the patient will continue taking a maintenance dose of 5 mg methimazole for a total of 6 months.

### Case 2

Twelve months later and after the patient had maintained a euthyroid state with negative TRAbs, a repeat CT scan was performed, which revealed total regression of the mass seen on the previous CT (Fig. 2C and 2D).

## Discussion

TH is rare outside adolescence so persistence of thymic tissue into adulthood may raise suspicion of thymoma, lymphoma, or metastatic tumors, among other diseases. Therefore, the detection of an anterior mediastinal mass usually leads to biopsy or even surgical removal. In GD and in the absence of any suspicious radiological feature, the diagnostic approach is different. TH seems to occur as a result of the GD, not as the cause. This is consistent with experimental studies in rats that have demonstrated thymic enlargement after administration of thyroid hormone and a reduction in thymic size after thyroidectomy [1]. Haider et al reviewed a total of 47 published case reports of GD-associated TH that had histologically proven TH or that showed regression on radiological follow-up, providing reassurance of the benign nature of TH associated with GD [2]. In this context, no interventional diagnostic or aggressive therapeutic procedure is usually needed, as shown in all cases described in the literature. The presence or absence of such an association is crucial to know in these patients to prevent unnecessary thymic evaluation and surgery.

TH in patients with GD was first described in 1914 [3]. Several reports of TH in GD have been published since then [4]. Nonetheless, the association is underestimated, as clinicians do not search for it. Considering the intrathoracic location of the thymus, TH is not often detected on a cervical ultrasound exam but is usually detected on a thoracic CT scan performed for reasons other than hyperthyroidism. For this reason, hyperplasia in the setting of GD is probably underdiagnosed.

Since TH is a known manifestation of GD, no further investigations of the thymic mass were conducted in patient 2, and thymic biopsy was not performed. Patient 1 illustrates the importance of expecting the GD-associated TH to regress with GD treatment. Thymectomy had already been performed to exclude thymoma. TH represents a diagnostic and therapeutic challenge for surgeons, and it is challenging to predict its pathologic diagnosis on the basis of imaging findings alone. It is true that PET-CT was not performed in the hospital of origin and could have been an option, as a hyperplastic thymus would demonstrate only mild uptake of fluorodeoxyglucose compared to the marked uptake by malignancies. However, more available evidence suggests that PET-CT has only marginal utility in differentiating benign from malignant processes within the thymus because the thymus can demonstrate normal physiologic uptake. This patient illustrates the importance of anticipating that the GD-associated TH will regress with GD treatment.

Regarding the histological pattern, 2 different forms of TH have been described in association with GD, lymphoid thymic hyperplasia (LTH) and true thymic hyperplasia (TTH), which arise via autoimmune mechanisms and excess thyroid hormones, respectively. LTH refers to an increase in lymphoid follicles and germinal centers [2] and is correlated with the immune activity underlying GD and other immunologically mediated disorders, including systemic lupus erythematosus, rheumatoid arthritis, and myasthenia gravis. It may or may not be associated with the enlargement of the thymus. By contrast, TTH always involves diffuse cortical and medullary parenchyma expansion. It presents with an enlarged thymus gland usually detected by chest tomography and seems to be due to a hyperthyroid state [2], thyroid hormone–inducing TTH via stimulation of the thymic cortex. Both TTH and LTH can coexist in the same gland. Unfortunately, most published case reports do not reference the histological definition of TH.

Therefore, although the causative mechanism of GD-associated TH has remained enigmatic, TH in the setting of GD seems to involve both immunological pathogenesis and thyroid hormone dependence.

First, an immunology-based pathogenesis of TH has been suggested. GD is an autoimmune disease characterized by the presence of agonistic TRAbs. It is known that these antibodies are responsible, by stimulating the TSHR, for hyperthyroidism, increased thyroid size, and extrathyroidal manifestations. The TSHR is expressed on the plasma membrane of thyroid follicular cells and plays a central role in the regulation of their function and growth. It is important to note that TSHR expression in extrathyroidal tissues (retro-orbital and pretibial fibroblasts) is higher in patients with GD than in patients without this condition. TSHR is expressed in thymic rat epithelial cells [5], and the expression of the TSHR in thymic tissue has been reported in multiple studies. The level of TSHR expression in the thymus was found to be between 5- and 50-fold lower than that in the thyroid [5]. In this context, Marín-Sánchez et al analyzed the relative expression of full-length human TSHR transcripts from DNA thymus and thyroid samples. The level of full-length TSHR expression in the thymus was higher than expected, at 20% of the level observed in the thyroid [6]. It is worth mentioning that although the level of TSHR expression in the thymus is lower than that in the thyroid, it can still be significant, considering that the thymus is where the T-lymphocyte repertoire is configured [6]. The function of TSHR in thymocytes has recently been analyzed [5]. TSHR gene expression in the thymus is strictly linked to the development of T cells within the thymus. Tolerance to thyroid autoantigens such as TSHR is achieved by exposing T lymphocytes to thyroid-specific antigens expressed in the thymus, thereby inducing anergy to these antigens. It is important to emphasize that TSHR gene expression in the thymus is linked to the maturation process of thymocytes, as suggested by Giménez-Barcons et al [7]. TRAbs could stimulate thymocytes through TSHR, explaining GD-associated TH.

Alternatively, it is known that thyroid hormones exert an influence on thymic growth. Höhn reported that T4 increased the thickness of the cortex and vascularity of both the cortex and medulla of the thymus [8]. Fabris et al measured the circulating thymic factor called thymulin in hyperthyroid and hypothyroid patients, which enhances the proliferation of T lymphocytes. Thymulin levels were higher in hyperthyroid patients than in normal individuals, whereas hypothyroid patients had lower thymulin levels than normal individuals [9]. This study may support the hypothesis that thyroid status influences thymic function by modulating thymic hormone levels. Murakami et al studied thymus size and density in 23 patients with GD. After treatment with antithyroid drugs, both thymus size and density decreased significantly [10].

With regard to time to regression of the thymic mass, as reported in the literature [1], a reduction in thymic size may take place with an average of 6 months and over 2 years. Thymic biopsy should be considered only in the presence of any suspicious radiological feature or if there is a lack of regression of the mass after achieving euthyroid status.

As a final thought, based on our illustrative cases and as previously reported in the literature, we believe that clinicians should be aware of the GD/TH association and the appropriate management to follow: The benign evolution as evidenced by regression of TH after resolution of the hyperthyroidism in all cases described in the literature supports a conservative approach that includes radiological follow-up, not surgical treatment.

## Learning Points

- There is a nonincidental and well-documented association GD and TH. This association is often underdiagnosed and unrecognized in routine clinical practice.
- Thymic regression with the resolution of hyperthyroidism is characteristic.
- Two different forms of TH have been described in association with GD: LTH and TTH.
- TH in the setting of GD seems to involve both an immunological pathogenesis and a thyroid hormone-dependent process.
- The benign evolution, as evidenced by regression of TH after resolution of hyperthyroidism, supports a conservative approach including radiological follow-up. This feature is crucial to be aware of to prevent unnecessary thymic evaluation and surgery.

## Contributors

All authors listed in the manuscript have contributed to the work: B.P.P. and P.A.G. contributed to the preparation of the manuscript and to the patient's clinical care; F.J.M.R. and A.A.M.A. contributed to revision and final approval of the manuscript.

## Data Availability

Data sharing is not applicable to this article as no data sets were generated or analyzed during the current study.

## Abbreviations

CT: computed tomography
FT4: free thyroxine
GD: Graves disease
LTH: lymphoid thymic hyperplasia
PET: positron emission tomography
TH: thymic hyperplasia
TRAbs: autoantibodies against the thyrotropin receptor
TSH: thyrotropin
TSHR: thyrotropin receptor
TTH: true thymic hyperplasia
